# Chlorido[2-meth­oxy-6-(2-pyridyl­methyl­imino­meth­yl)phenolato]zinc(II)

**DOI:** 10.1107/S1600536809037015

**Published:** 2009-10-03

**Authors:** Ning Sheng

**Affiliations:** a Department of Chemistry & Chemical Engineering, Jining University, Qufu 273155, People’s Republic of China

## Abstract

In the title mol­ecule, [Zn(C_14_H_13_N_2_O_2_)Cl], the Zn(II) ion is coordinated by one O and two N atoms from the Schiff base ligand, and a chloride anion in a distorted square-planar geometry. In the crystal structure, π–π inter­actions link the approximately planar (mean deviation 0.0569 Å) mol­ecules into stacks parallel to the *a* axis.

## Related literature

For properties of transition metal complexes with Schiff base ligands, see: Ghosh *et al.* (2006 ▶); Singh *et al.* (2007 ▶); Ward (2007 ▶). For details of the synthesis of the ligand, see Kannappan *et al.* (2005 ▶). For related structures, see: Li & Zhang (2004 ▶); Chen (2005 ▶); You (2005 ▶).
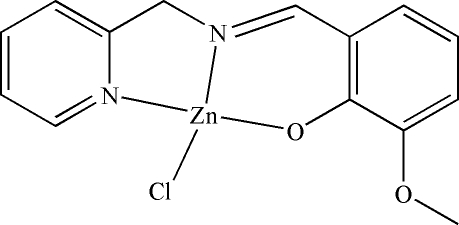


## Experimental

### 

#### Crystal data

[Zn(C_14_H_13_N_2_O_2_)Cl]
*M* *_r_* = 342.08
Monoclinic, 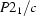

*a* = 7.1013 (5) Å
*b* = 18.2673 (14) Å
*c* = 10.3241 (8) Å
β = 104.789 (1)°
*V* = 1294.89 (17) Å^3^
*Z* = 4
Mo *K*α radiation
μ = 2.10 mm^−1^
*T* = 293 K
0.31 × 0.25 × 0.23 mm

#### Data collection

Bruker APEXII CCD area-detector diffractometer
Absorption correction: multi-scan (*SADABS*; Sheldrick, 2003 ▶) *T*_min_ = 0.562, *T*_max_ = 0.643
6845 measured reflections
2538 independent reflections
2263 reflections with *I* > 2σ(*I*)
*R*_int_ = 0.017

#### Refinement

*R*[*F* ^2^ > 2σ(*F* ^2^)] = 0.027
*wR*(*F* ^2^) = 0.082
*S* = 1.06
2538 reflections
182 parameters
H-atom parameters constrained
Δρ_max_ = 0.36 e Å^−3^
Δρ_min_ = −0.31 e Å^−3^

### 

Data collection: *APEX2* (Bruker, 2004 ▶); cell refinement: *SAINT-Plus* (Bruker, 2001 ▶); data reduction: *SAINT-Plus*; program(s) used to solve structure: *SHELXS97* (Sheldrick, 2008 ▶); program(s) used to refine structure: *SHELXL97* (Sheldrick, 2008 ▶); molecular graphics: *XP* (Sheldrick, 1998 ▶); software used to prepare material for publication: *XP*.

## Supplementary Material

Crystal structure: contains datablocks global, I. DOI: 10.1107/S1600536809037015/cv2598sup1.cif

Structure factors: contains datablocks I. DOI: 10.1107/S1600536809037015/cv2598Isup2.hkl

Additional supplementary materials:  crystallographic information; 3D view; checkCIF report

## Figures and Tables

**Table:** 

Zn1—O1	1.9059 (16)
Zn1—N2	1.9288 (18)
Zn1—N1	2.0112 (19)
Zn1—Cl1	2.2373 (6)

**Table:** 

*Cg*1⋯*Cg*2^i^	3.566 (4)
*Cg*1⋯*Cg*2^ii^	3.767 (7)

Symmetry codes: (i) 
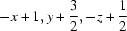
; (ii) 
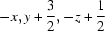
. *Cg*1 and *Cg*2 are centroids of atoms C1–C6 and N1/C9–C13, respectively.

**Fig. 1.**
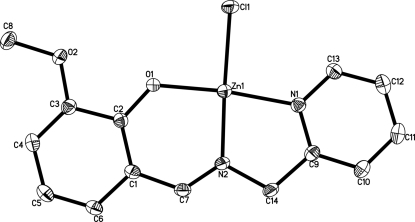
View of (I) with the atom-labelling scheme. Displacement ellipsoids are drawn at the 30% probability level. All H-atoms were omitted for clarity.

## supplementary crystallographic information

### Comment

Transition metal-Schiff based complexes have been intensively focused on owing to their excellent physical and chemical properties including magnetic, optics and catalysis (Ghosh *et al.*, 2006; Singh *et al.*, 2007; Ward *et al.*, 2007). Additionally, their intriguing biological activites also attract a lot of attentions. Herein, we report the structure of a new zinc complex with asymmetric tridentate Schiff base ligand.

In the title compound, (I) (Fig. 1), the Zn(II) ion is four coordinated with a slightly distorted square planar coordination sphere formed by two N atoms and one O atom from the asymmetric tridentate Schiff base ligand, and the fourth position is occupied by one Cl anion. The mean deviation of the plane formed by ZnN_2_OCl unit is 0.0569 Å. The Zn—O, Zn—N and Zn—Cl bond lengths are all comparable to those found in other Zn Schiff base complexes (You, 2005; Chen, 2005; Li, *et al.*, 2004). It is worthing noting that the asymmetric unit can be linked into one-dimensional supermolecular structure *via* the π···π interactions (Table 1).

### Experimental

The Schiff base was synthesized according to the literature method (Kannappan *et al.*, 2005). The synthesis of the title complex was carried out by reacting ZnCl_2_ and the schiff-base ligand (1:1, molar ratio) in methanol under the refelux condition. The cooled solution was filtrated and left for slow evaperation in air to obtain single-crystal suitable for X-ray diffraction.

### Refinement

All H atoms were geometrically positioned (C—H = 0.93 - 0.96 Å) and refined as riding, with *U*_iso_(H) = 1.2*U*_eq_(C).

### Crystal data

**Table:**

[Zn(C_14_H_13_N_2_O_2_)Cl]	*F*(000) = 696
*M**_r_* = 342.08	*D*_x_ = 1.755 Mg m^−^^3^
Monoclinic, *P*2_1_/*c*	Mo *K*α radiation, λ = 0.71073 Å
*a* = 7.1013 (5) Å	Cell parameters from 3850 reflections
*b* = 18.2673 (14) Å	θ = 3.0–26.0°
*c* = 10.3241 (8) Å	µ = 2.10 mm^−^^1^
β = 104.789 (1)°	*T* = 293 K
*V* = 1294.89 (17) Å^3^	Block, colourless
*Z* = 4	0.31 × 0.25 × 0.23 mm

### Data collection

**Table:**

Bruker APEXII CCD area-detector diffractometer	2538 independent reflections
Radiation source: fine-focus sealed tube	2263 reflections with *I* > 2σ(*I*)
graphite	*R*_int_ = 0.017
φ and ω scans	θ_max_ = 26.0°, θ_min_ = 2.2°
Absorption correction: multi-scan (*SADABS*; Sheldrick, 2003)	*h* = −8→8
*T*_min_ = 0.562, *T*_max_ = 0.643	*k* = −18→22
6845 measured reflections	*l* = −12→10

### Refinement

**Table:**

Refinement on *F*^2^	Primary atom site location: structure-invariant direct methods
Least-squares matrix: full	Secondary atom site location: difference Fourier map
*R*[*F*^2^ > 2σ(*F*^2^)] = 0.027	Hydrogen site location: inferred from neighbouring sites
*wR*(*F*^2^) = 0.082	H-atom parameters constrained
*S* = 1.06	*w* = 1/[σ^2^(*F*_o_^2^) + (0.0487*P*)^2^ + 0.6563*P*] where *P* = (*F*_o_^2^ + 2*F*_c_^2^)/3
2538 reflections	(Δ/σ)_max_ = 0.006
182 parameters	Δρ_max_ = 0.36 e Å^−^^3^
0 restraints	Δρ_min_ = −0.31 e Å^−^^3^

### Special details

**Table:**

Geometry. All e.s.d.'s (except the e.s.d. in the dihedral angle between two l.s. planes) are estimated using the full covariance matrix. The cell e.s.d.'s are taken into account individually in the estimation of e.s.d.'s in distances, angles and torsion angles; correlations between e.s.d.'s in cell parameters are only used when they are defined by crystal symmetry. An approximate (isotropic) treatment of cell e.s.d.'s is used for estimating e.s.d.'s involving l.s. planes.
Refinement. Refinement of *F*^2^ against ALL reflections. The weighted *R*-factor *wR* and goodness of fit *S* are based on *F*^2^, conventional *R*-factors *R* are based on *F*, with *F* set to zero for negative *F*^2^. The threshold expression of *F*^2^ > σ(*F*^2^) is used only for calculating *R*-factors(gt) *etc*. and is not relevant to the choice of reflections for refinement. *R*-factors based on *F*^2^ are statistically about twice as large as those based on *F*, and *R*- factors based on ALL data will be even larger.

### Fractional atomic coordinates and isotropic or equivalent isotropic displacement parameters (Å^2^)

**Table:**

	*x*	*y*	*z*	*U*_iso_*/*U*_eq_	
Zn1	0.33329 (4)	0.484815 (14)	0.11005 (3)	0.02880 (12)	
Cl1	0.45273 (10)	0.40303 (3)	0.27143 (6)	0.04062 (17)	
O1	0.2046 (2)	0.40698 (8)	−0.00080 (16)	0.0324 (4)	
O2	0.0568 (3)	0.28355 (9)	−0.10946 (17)	0.0389 (4)	
N1	0.4516 (3)	0.57415 (10)	0.21313 (18)	0.0272 (4)	
N2	0.2579 (3)	0.55672 (10)	−0.03049 (19)	0.0276 (4)	
C1	0.0718 (3)	0.47592 (12)	−0.2000 (2)	0.0282 (5)	
C2	0.1050 (3)	0.41075 (12)	−0.1239 (2)	0.0265 (5)	
C3	0.0236 (3)	0.34459 (12)	−0.1884 (2)	0.0295 (5)	
C4	−0.0793 (3)	0.34472 (14)	−0.3194 (2)	0.0334 (5)	
H4	−0.1296	0.3011	−0.3605	0.040*	
C5	−0.1094 (3)	0.40998 (15)	−0.3922 (2)	0.0356 (5)	
H5	−0.1803	0.4094	−0.4814	0.043*	
C6	−0.0367 (3)	0.47439 (13)	−0.3345 (2)	0.0325 (5)	
H6	−0.0588	0.5175	−0.3840	0.039*	
C7	0.1495 (3)	0.54479 (13)	−0.1478 (2)	0.0303 (5)	
H7	0.1183	0.5850	−0.2046	0.036*	
C8	−0.0093 (4)	0.21497 (13)	−0.1699 (3)	0.0433 (6)	
H8A	−0.1460	0.2180	−0.2128	0.065*	
H8B	0.0128	0.1776	−0.1024	0.065*	
H8C	0.0608	0.2032	−0.2353	0.065*	
C9	0.4324 (3)	0.63695 (12)	0.1444 (2)	0.0281 (5)	
C10	0.5065 (3)	0.70331 (13)	0.2015 (3)	0.0340 (5)	
H10	0.4936	0.7456	0.1499	0.041*	
C11	0.5983 (4)	0.70499 (14)	0.3345 (3)	0.0388 (6)	
H11	0.6496	0.7484	0.3760	0.047*	
C12	0.6133 (4)	0.64061 (15)	0.4060 (3)	0.0393 (6)	
H12	0.6722	0.6406	0.4974	0.047*	
C13	0.5419 (3)	0.57673 (14)	0.3432 (2)	0.0338 (5)	
H13	0.5569	0.5337	0.3929	0.041*	
C14	0.3226 (4)	0.63242 (12)	0.0026 (2)	0.0325 (5)	
H14A	0.2104	0.6646	−0.0133	0.039*	
H14B	0.4045	0.6481	−0.0543	0.039*	

### Atomic displacement parameters (Å^2^)

**Table:**

	*U*^11^	*U*^22^	*U*^33^	*U*^12^	*U*^13^	*U*^23^
Zn1	0.03695 (18)	0.02365 (17)	0.02486 (17)	0.00029 (10)	0.00615 (12)	0.00196 (10)
Cl1	0.0635 (4)	0.0280 (3)	0.0268 (3)	0.0019 (3)	0.0049 (3)	0.0074 (2)
O1	0.0444 (9)	0.0229 (8)	0.0246 (8)	−0.0019 (7)	−0.0010 (7)	0.0024 (6)
O2	0.0494 (10)	0.0228 (8)	0.0374 (9)	−0.0037 (7)	−0.0017 (8)	−0.0012 (7)
N1	0.0300 (9)	0.0251 (9)	0.0270 (9)	0.0010 (7)	0.0082 (8)	0.0000 (8)
N2	0.0343 (10)	0.0211 (9)	0.0271 (9)	0.0011 (8)	0.0073 (8)	0.0032 (7)
C1	0.0271 (11)	0.0309 (12)	0.0257 (11)	0.0035 (9)	0.0051 (9)	0.0031 (9)
C2	0.0238 (10)	0.0285 (11)	0.0263 (11)	0.0013 (9)	0.0051 (8)	−0.0008 (9)
C3	0.0291 (11)	0.0269 (11)	0.0322 (12)	0.0013 (9)	0.0076 (9)	−0.0007 (9)
C4	0.0316 (12)	0.0368 (13)	0.0306 (12)	−0.0017 (10)	0.0057 (10)	−0.0078 (10)
C5	0.0311 (12)	0.0480 (15)	0.0250 (12)	0.0023 (11)	0.0022 (9)	−0.0006 (10)
C6	0.0336 (12)	0.0365 (13)	0.0256 (11)	0.0040 (10)	0.0043 (10)	0.0052 (10)
C7	0.0356 (12)	0.0272 (12)	0.0273 (11)	0.0044 (9)	0.0069 (9)	0.0067 (10)
C8	0.0450 (15)	0.0260 (12)	0.0523 (16)	−0.0036 (10)	0.0002 (12)	−0.0053 (11)
C9	0.0294 (11)	0.0247 (11)	0.0322 (12)	0.0003 (9)	0.0119 (9)	−0.0015 (9)
C10	0.0365 (12)	0.0254 (11)	0.0415 (13)	−0.0019 (10)	0.0128 (10)	−0.0028 (10)
C11	0.0344 (12)	0.0363 (13)	0.0463 (14)	−0.0088 (11)	0.0116 (11)	−0.0129 (12)
C12	0.0348 (12)	0.0480 (15)	0.0338 (13)	−0.0062 (11)	0.0062 (10)	−0.0084 (11)
C13	0.0354 (12)	0.0358 (12)	0.0286 (12)	−0.0022 (10)	0.0052 (10)	0.0017 (10)
C14	0.0490 (14)	0.0183 (10)	0.0304 (12)	0.0009 (9)	0.0106 (10)	0.0022 (9)

### Geometric parameters (Å, °)

**Table:**

Zn1—O1	1.9059 (16)	C5—C6	1.360 (4)
Zn1—N2	1.9288 (18)	C5—H5	0.9300
Zn1—N1	2.0112 (19)	C6—H6	0.9300
Zn1—Cl1	2.2373 (6)	C7—H7	0.9300
O1—C2	1.289 (3)	C8—H8A	0.9600
O2—C3	1.366 (3)	C8—H8B	0.9600
O2—C8	1.425 (3)	C8—H8C	0.9600
N1—C13	1.333 (3)	C9—C10	1.391 (3)
N1—C9	1.337 (3)	C9—C14	1.475 (3)
N2—C7	1.277 (3)	C10—C11	1.362 (4)
N2—C14	1.470 (3)	C10—H10	0.9300
C1—C6	1.406 (3)	C11—C12	1.378 (4)
C1—C2	1.412 (3)	C11—H11	0.9300
C1—C7	1.423 (3)	C12—C13	1.368 (4)
C2—C3	1.429 (3)	C12—H12	0.9300
C3—C4	1.363 (3)	C13—H13	0.9300
C4—C5	1.396 (4)	C14—H14A	0.9700
C4—H4	0.9300	C14—H14B	0.9700
			
Cg1···Cg2^i^	3.566 (4)	Cg1···Cg2^ii^	3.767 (7)
			
O1—Zn1—N2	93.32 (7)	C1—C6—H6	120.0
O1—Zn1—N1	174.02 (7)	N2—C7—C1	126.3 (2)
N2—Zn1—N1	81.01 (8)	N2—C7—H7	116.9
O1—Zn1—Cl1	88.94 (5)	C1—C7—H7	116.9
N2—Zn1—Cl1	173.99 (6)	O2—C8—H8A	109.5
N1—Zn1—Cl1	96.89 (6)	O2—C8—H8B	109.5
C2—O1—Zn1	127.63 (14)	H8A—C8—H8B	109.5
C3—O2—C8	117.96 (19)	O2—C8—H8C	109.5
C13—N1—C9	117.5 (2)	H8A—C8—H8C	109.5
C13—N1—Zn1	126.18 (16)	H8B—C8—H8C	109.5
C9—N1—Zn1	116.30 (15)	N1—C9—C10	123.1 (2)
C7—N2—C14	117.25 (19)	N1—C9—C14	115.78 (19)
C7—N2—Zn1	125.56 (16)	C10—C9—C14	121.1 (2)
C14—N2—Zn1	117.11 (14)	C11—C10—C9	118.6 (2)
C6—C1—C2	120.3 (2)	C11—C10—H10	120.7
C6—C1—C7	117.0 (2)	C9—C10—H10	120.7
C2—C1—C7	122.7 (2)	C10—C11—C12	118.2 (2)
O1—C2—C1	124.4 (2)	C10—C11—H11	120.9
O1—C2—C3	118.0 (2)	C12—C11—H11	120.9
C1—C2—C3	117.6 (2)	C13—C12—C11	120.4 (2)
C4—C3—O2	124.1 (2)	C13—C12—H12	119.8
C4—C3—C2	120.8 (2)	C11—C12—H12	119.8
O2—C3—C2	115.12 (19)	N1—C13—C12	122.2 (2)
C3—C4—C5	120.4 (2)	N1—C13—H13	118.9
C3—C4—H4	119.8	C12—C13—H13	118.9
C5—C4—H4	119.8	N2—C14—C9	109.77 (18)
C6—C5—C4	120.9 (2)	N2—C14—H14A	109.7
C6—C5—H5	119.5	C9—C14—H14A	109.7
C4—C5—H5	119.5	N2—C14—H14B	109.7
C5—C6—C1	120.1 (2)	C9—C14—H14B	109.7
C5—C6—H6	120.0	H14A—C14—H14B	108.2

Symmetry codes: (i) −*x*+1, *y*+3/2, −*z*+1/2; (ii) −*x*, *y*+3/2, −*z*+1/2.
